# Failure of intrathecal allogeneic mesenchymal stem cells to halt progressive demyelination in two boys with cerebral adrenoleukodystrophy

**DOI:** 10.1002/sctm.19-0304

**Published:** 2020-02-05

**Authors:** Ashish Gupta, Paul J. Orchard, Weston P. Miller, Dave R. Nascene, Gerald V. Raymond, Daniel J. Loes, David H. McKenna, Troy C. Lund

**Affiliations:** ^1^ Division of Pediatric Blood and Marrow Transplant University of Minnesota Minneapolis Minnesota; ^2^ Sangamo Therapeutics Richmond California; ^3^ Department of Diagnostic Radiology University of Minnesota Minneapolis Minnesota; ^4^ Department of Neurology Johns Hopkins Medicine Baltimore Maryland; ^5^ Department of Laboratory Medicine and Pathology, Transfusion Medicine University of Minnesota Minneapolis Minnesota

**Keywords:** adrenoleukodystrophy, autoimmune, demyelination, inflammation, mesenchymal stem cells

## Abstract

Cerebral adrenoleukodystrophy is an inflammatory demyelinating condition that is the result of a mutation in the X‐linked *ABCD1* gene, a peroxisomal very long chain fatty acid transporter. Although mutations in this gene result in adrenal insufficiency in the majority of affected individuals, 40% of those affected develop the demyelinating cerebral form, cerebral adrenoleukodystrophy (CALD). CALD is characterized by imaging findings of demyelination and contrast enhancement on magnetic resonance imaging (MRI). Although allogeneic hematopoietic cell transplantation can arrest progression of CALD early in its course, there is no accepted therapy for patients with advanced CALD. Mesenchymal stem cells (MSCs) have been used in a variety of clinical trials to capitalize on their anti‐inflammatory properties as well as promote tissue repair. We delivered MSCs via intrathecal (IT) route to two boys with rapidly advancing CALD. The first boy received three doses 1 week apart, whereas the second boy received a single dose of IT MSCs. We note delivery of IT MSCs was feasible and without complication. Follow‐up MRI scans after IT MSC delivery showed progressive demyelination in the first patient and no change in demyelination or contrast enhancement in the second patient. Although the infusion of IT MSCs was safe, it did not halt CALD progression in this setting, and future studies should focus on patient selection and dose optimization.

## Graphical abstract

Mesenchymal stem cells were delivered intrathecally to attenuate cerebral inflammation in two boys with advanced cerebral adrenoleukodystrophy. Scale bar represent 100 μm.

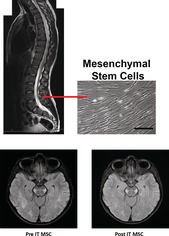

**Lessons learned**

- Mesenchymal stem cells can be safely delivered to boys with cerebral adrenoleukodystrophy.
- Advanced cerebral adrenoleukodystrophy may not respond to intrathecal injection.

**Significance statement**

Cerebral adrenoleukodystrophy (CALD) is characterized by imaging findings of demyelination, inflammation, and contrast enhancement on magnetic resonance imaging (MRI). There is no accepted therapy for patients with advanced cerebral adrenoleukodystrophy. Mesenchymal stem cells (MSCs) were delivered via intrathecal (IT) route to two boys with rapidly advancing CALD in hopes of utilizing their anti‐inflammatory ability to halt disease progression. The delivery of IT MSC was feasible and without complication, although follow‐up MRI scans after IT MSC delivery showed progressive demyelination in both patients. This may have been due to the advanced nature of disease in the patients or an inadequate cell dose.

## 1 INTRODUCTION

Adrenoleukodystrophy protein (ALDP) is a transporter responsible for the movement of acylated very long chain fatty acids (VLCFAs) into peroxisomes in all tissues, facilitating degradation by beta‐oxidation. Adrenoleukodystrophy (ALD) results from the lack of ALDP expression due to mutations in the *ABCD1* gene located on the X‐chromosome, resulting in VLCFA accumulation in the peripheral tissues including the central nervous system. The adrenal gland is exquisitely sensitive to VLCFA buildup for unknown reasons and undergoes deterioration leading to adrenal insufficiency in most males with adrenoleukodystrophy.^1^, ^2^, ^3^

In 30%‐40% of boys with ALD, a neuroinflammatory process known as cerebral ALD (CALD) is initiated between the ages of 4 and 10 years. The trigger and pathophysiological mechanism surrounding this process are largely unknown. On magnetic resonance imaging (MRI), CALD is denoted by signs of demyelination (changes in signal on T2‐weighted imaging) accompanied by blood‐brain barrier (BBB) disruption defined by intravenous contrast enhancement positivity indicating an active disease process. Both microglial cell death and endothelial disruption contribute to the pathophysiology of active disease.^4^, ^5^ The origination of the inflammatory process that accompanies CALD is unclear, as is how inflammation contributes to the overall neuropathology.^6^ The neuroinflammation is characterized by activated microglial cells and invading immune cells such T cells and macrophages, with the occasional B cells and immunoglobulin G‐expressing plasma cells.^7^ Like other inflammatory conditions, the presence of a humoral system response and auto‐antibody production has also been reported.^8^ Only early hematopoietic cell transplant (HCT) can arrest the cerebral disease process in CALD via unclear mechanisms but may involve immune modulation and healthy donor macrophage/microglial engraftment.^9^ Mesenchymal stem cells are a stroma cell type isolated most commonly from the bone marrow, although similar cell types are found associated with many organ systems.^10^ MSCs have been demonstrated to have a wide variety of tissue repair‐ and cell growth‐promoting properties and have also been extensively studied clinically for their anti‐inflammatory properties in a wide variety of conditions, with improvements observed in select patients.^10^

Given that CALD has a significant neuroinflammatory component, we reasoned that MSCs may be able to counter cerebral inflammation and slow disease progression. Although prior studies have used both auto and allogeneic MSCs to treat neurological disease,^11^ we chose an allogeneic approach being that CALD is caused by a single gene defect that would be present in an autologous MSC product and perhaps dampen its therapeutic potential. In this study, we present two clinical cases in which MSCs were delivered intrathecally to boys with advanced CALD and thus not eligible for HCT, with the goal of arresting disease progression as assessed radiologically.

## 2 MATERIALS AND METHODS

**Figure 1:**
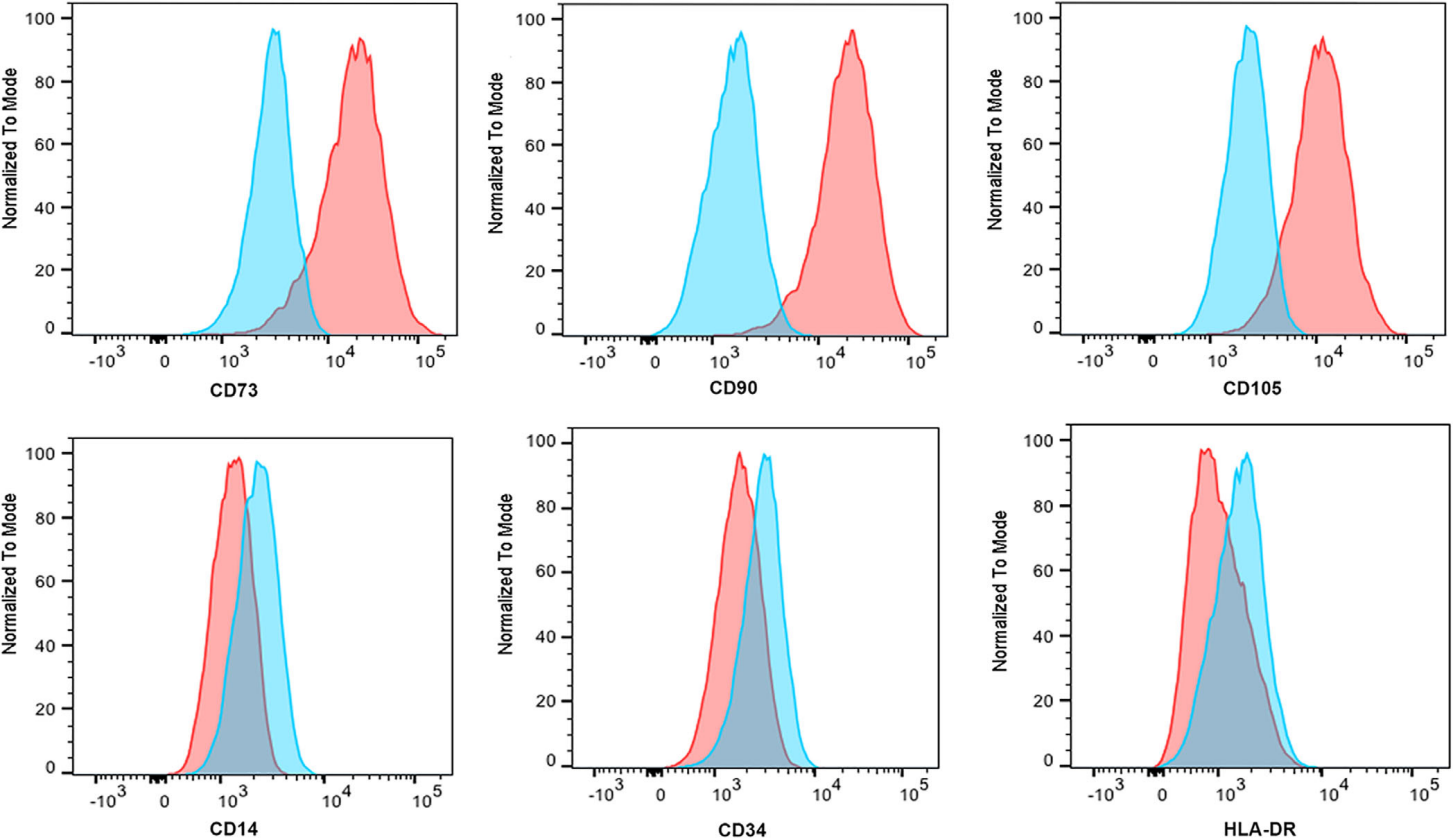
Flow cytometry of third‐party mesenchymal stem cells (MSCs). Shown are representative histograms for MSC‐related cell surface markers on the infused cell product prior to administration. Gating was set to exclude debris and doublets prior to specific marker discrimination. Red histograms indicate the specific antibody compared with the isotype control in blue. The bottom three histograms show markers typically absent on MSCs. CD19 was also tested and was not expressed by MSCs (data not shown)

Enrichment of the mononuclear cell fraction of the marrow was accomplished using a semiautomated separation method involving ficoll hypaque density gradient medium, specific gravity 1.077 g/dL (Isolymph; Gallard‐Schlesinger Industries, Carle Place, NY) followed by washing with Hank's Balanced Salt Solution (without phenol red, calcium, or magnesium). Cells were seeded at 1.0‐1.5 × 10^5^ cells/cm^2^ at a media depth of 1.6 mm in an appropriately sized culture vessel and placed in a 5% CO_2_ incubator at 37°C. Growth media consisted of alpha‐minimal essential medium, 16.5% fetal bovine serum, and L‐glutamine (2 mM). On days 1 and 2 after plating, nonadherent cells were removed and the media changed. Fresh media was exchanged every 2‐4 days until approximately 70%‐80% confluency was reached. The cells were washed with Dulbecco's phosphate‐buffered saline (DPBS), detached via trypsin, and inoculated into a cell factory at 40‐50 cells/cm^2^, exchanging the media every 2‐4 days. At approximately 70%‐80% confluence, cells were washed with DPBS, harvested, resuspended in 5% human serum albumin (HSA) at 2‐20 × 10^6^ cells/mL (2× final freeze concentration), and cryopreserved at a 1:1 ratio with a freezing solution consisting of 60% Plasmalyte‐A, 20% dimethyl sulfoxide, and 20% HSA (reagents were at 2× final freeze concentration). Prior to injection, MSCs were thawed, washed in DPBS, and then resuspended in Elliott's B Solution (Lukare Medical, LLC, Scotch Plains, New Jersey). Lot release criteria included 14‐day sterility testing, normal karyotyping, flow cytometry, mycoplasma negativity by polymerase chain reaction, and <5.0 EU/kg/total product endotoxin levels. For flow cytometry analysis, all debris was excluded using forward vs side scatter. All events minus debris were then analyzed for CD14, CD19, CD34, CD45, CD73, CD90, and CD105. Expanded cells showed cell marker expression consistent with MSCs (Figure 1).^12^ This study was approved by the Committee on the Use of Human Subjects in Research at the University of Minnesota. Informed consent was obtained from all participants and/or their legal guardian/s.

## 3 RESULTS

### 3.1 Case reports

**Figure 2:**
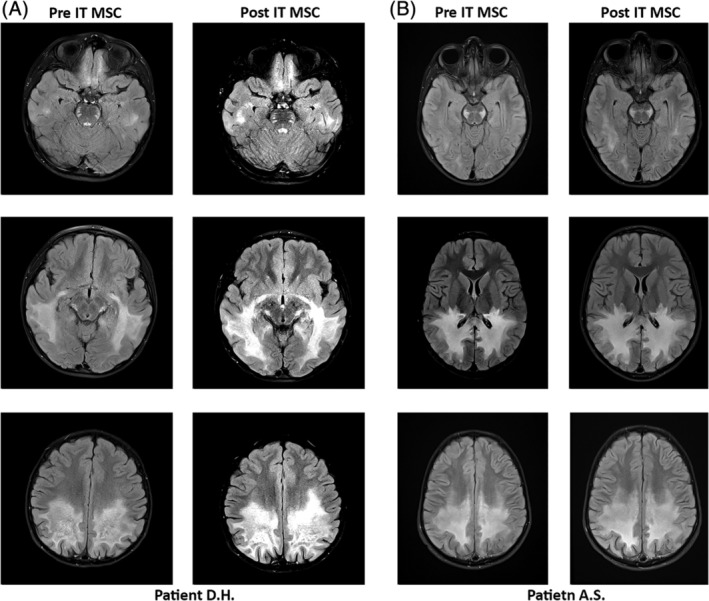
Magnetic resonance images (MRIs) before and after mesenchymal stem cells (MSCs). A, Patient D.H. MRI was 2 months following the third intrathecal (IT) MSC dose. B, Patient A.S. MRI was 1 month after the dose of IT MSCs

D.H. was a 5‐year‐old male who came to medical attention following teacher and parental concerns for declining visual and auditory processing function. Brain MRI revealed diffuse white matter abnormalities indicating extensive demyelination within the parietooccipital white matter and corpus callosum associated with intravenous contrast enhancement. Plasma VLCFA analysis showed abnormalities pathognomonic of ALD, and adrenal function testing revealed insufficiency. The radiographic MRI score measuring CALD disease severity was 12.5.^13^ Neurologic function, measured using the CALD neurologic functional score (NFS) paradigm (supplemental online Table 1), was 3 (having visual, auditory, and motor involvement). The patient was deemed too advanced to undergo standard HCT. As an alternative therapy, he received three weekly doses of third‐party MSCs via intrathecal (IT) injection at a dose of 5 × 10^6^ cells/kg for each injection. Premedications were given prior to MSC infusion and included stress dose hydrocortisone, weight‐based dosing of acetaminophen, and diphenhydramine. Two months after the third IT injection, an MRI was performed that again showed demyelination in white matter including the corpus callosum along with increased demyelination in the posterior frontal lobes. Persistent restricted diffusion and mild gadolinium enhancement were also noted consistent with disease progression (Figure 2A). Cerebrospinal fluid (CSF) was evaluated before MSC therapy and showed a total protein of 53 mg/dL without pleocytosis. Follow‐up CSF studies for the following 2 weeks and sampling at 2 months after MSC therapy showed no change in any parameter.

A.S. was a 7‐year‐old boy who had normal growth, development, and cognitive skills until 6 months prior to his diagnosis when he presented with difficulty completing math assignments (that had previously been easy for him) and developed difficulties following instructions in school. He also had spatial disorientation and balance and gait disturbances. Further assessments included an MRI that showed extensive demyelination within the white matter including the ventral pons along the corticospinal tracts and corpus callosum, accompanied by abnormal gadolinium contrast enhancement; the ALD MRI severity score was 17 (Figure 2B). Elevated VLCFAs and a mutation in the *ABCD1* gene confirmed the diagnosis of X‐linked CALD. His NFS was 7 (losses in vision, processing, and mobility; supplemental online Table 1). The patient received 5 × 10^6^/kg third‐party MSCs by IT route. For this patient, MSCs were thawed and cultured for 48 hours prior to IT delivery owing to a recent report that freshly thawed MSCs used in treatment may have impaired immunosuppressive activity compared with MSCs growing in the log phase used as therapy.^14^ Premedications were given prior to MSC infusion, similar to the previous patient. Additional cytogenetic analysis of the re‐expanding MSCs indicated a new clonal chromosomal abnormality had been acquired during the brief expansion, and although the first dose of MSCs was given, the latter two treatments were withheld. An MRI was performed 1 month after the IT MSC injection and showed unchanged demyelination signal and no change in the abnormal gadolinium contrast enhancement, indicating that his cerebral disease was not in remission (Figure 2B). In both of these cases, further treatment was not offered.

## 4 DISCUSSION

Multiple characteristics of MSCs include abilities to support hematopoiesis,^15^, ^16^ differentiate into numerous cell types for tissue regeneration,^17^ and modulate the immune response.^18^, ^19^ The effects on T cells include an enhanced T helper 2 response, with increased interleukin (IL)‐4 and IL‐10 production and increased T‐regulatory cells frequency, which appears independent of major histocompotbility complex disparity between donor and recipient.^20^, ^21^ Because CALD may be associated with degeneration, immune dysregulation, and inflammation, it is reasonable to consider investigating the biologic proprieties of MSCs for therapeutic potential.

Intrathecal MSCs have been used in the treatment of amyotrophic lateral sclerosis. Ho et al reported on 33 patients who received two doses (1 × 10^6^ cells/kg) of IT MSCs 1 month apart. Changes in the Amyotrophic Lateral Sclerosis Functional Rating Scale‐Revised score from baseline to 4 and 6 months after injection were significantly different from the non‐MSC‐treated group.^22^ There were also significant changes in CSF cytokines with increases in levels of transforming growth factor‐ beta 1 and decreases in monocyte chemoattractant protein‐1 in the treated individuals possibly indicating an anti‐inflammatory effect.^22^

Some studies have attempted to use MSCs differentiated toward a neural phenotype as treatment. A study treating patients with epilepsy with antiepileptic drugs plus autologous MSCs (1 × 10^6^ cell/kg followed by a single IT dose of neural‐induced MSCs at 0.1 × 10^6^ cells/kg) showed that some patients in the MSC group (compared with patients only receiving antiepileptic drugs) went into remission, although the sample size was small (n = 10 in the treatment group).^23^ In another small (n = 4) study in treating drug‐resistant patients with epilepsy with IT and intravenous MSCs, all patients showed a reduction in the number of epileptic seizures (from 10‐40 per week to 1 per week) and an absence of status epilepticus episodes (from 4 per week to 0 per week).^24^ Patients with multiple sclerosis have also been treated with IT neural progenitors derived from MSCs. In a small study (n = 20), Harris et al reported improvement in expanded disability status scale scores in 9 of the treated patients; although far from conclusive, this could indicate a potential benefit. These studies indicate that some patients have a therapeutic benefit from MSCs, although the mechanism of action is unclear and may vary depending on the condition being treated.

Although the patients in our study showed disease progression after MSCs, we cannot rule out the possibility that an inadequate MSC dose was used (and one patient only received a single dose), although anywhere from 0.1 to 10 × 10^6^ cells/kg are commonly found as doses in clinical trials.^10^ This potential variability in dosing limits conclusive interpretation of the optimal effect. An additional possibility is that these patients were far too advanced to receive benefit from intervention. We have previously shown that patients with very involved cerebral disease (MRI score > 10) do poorly after HCT.^25^ In any event, further novel therapies need to be identified for severely affected boys with CALD.

## 5 CONCLUSION

The delivery of MSCs intrathecally was safe and well tolerated. We did not find toxicity related to the delivery of the MSCs. Unfortunately, neither patient showed any reversal or arrest in the progression of disease as assessed by MRI studies. Whether this was because of the rapidly progressive nature of their disease or other factors such as lack of adequate cell dosing is unknown, and future studies should focus on these factors.

## CONFLICT OF INTEREST

D.R.N. declared advisory role with Biogen, WorldCare Clinical. G.R. declared consulting role with Bluebird bio and Retrophin. D.J.L. declared consulting role with Bluebird Bio with regard to MRI interpretation in patients with ALD. The other authors declared no potential conflicts of interest.

## AUTHOR CONTRIBUTIONS

A.G.: data analysis and interpretation, manuscript writing, final approval of manuscript; W.P.M.: manuscript editing, final approval of manuscript, provision of study material or patients; P.J.O.: conception and design; D.R.N.: provision of study material or patients, data analysis and interpretation; G.V.R.: provision of study material or patients; D.J.L.: data analysis and interpretation; D.H.M.: provision of study material or patients, collection and/or assembly of data, data analysis and interpretation; T.C.L.: conception and design, data analysis and interpretation, manuscript writing, final approval of manuscript.

## DATA AVAILABILITY STATEMENT

The data that support the findings of this study are available on request from the corresponding author.

## Supporting information


**Supplemental Table 1** Neurologic function score (NFS) in patients with adrenoleukodystrophy
