# Supplementary material for: Failure of intrathecal allogeneic mesenchymal stem cells to halt progressive demyelination in two boys with cerebral adrenoleukodystrophy
Source: Stem Cells Transl Med. 2020 Feb 5;9(5):554–8. doi: 10.1002/sctm.19-0304 (PMC7180290; doi:10.1002/sctm.19-0304)
Supplement: Supplementary file 1 — Supplemental Table 1 Neurologic function score (NFS) in patients with adrenoleukodystrophy [file SCT3-9-554-s001.docx]

| **Neurologic Function Score** | **Points** |
| --- | --- |
| Hearing/auditory processing problems | 1 |
| Aphasia/apraxia | 1 |
| Loss of communication | 3 |
| Vision impairment/fields cut | 1 |
| Cortical blindness | 2 |
| Swallowing difficulty or other central nervous system dysfunction | 2 |
| Tube feeding | 2 |
| Running difficulties/hyperreflexia | 1 |
| Walking difficulties/spasticity/spastic gait (no assistance) | 1 |
| Spastic gait (needs assistance) | 2 |
| Wheelchair required | 2 |
| No voluntary movement | 3 |
| Episodes of incontinency | 1 |
| Total incontinency | 2 |
| Nonfebrile seizures | 1 |
|  | 25 total |

**Supplemental Table 1**. The cerebral adrenoleukodystrophy neurologic function score (NFS) used to evaluate gross clinical neurologic severity for the cALD cohort pre-transplantation. Note that a score of zero denotes absence of clinical signs of cerebral disease. Maximal signs within a domain score the total of all grades within that domain (for example, a patient with “total urinary or fecal incontinency” scores 3, for the sum of “Episodes of incontinency” [1 point] and “Total Incontinency” [2 additional points]).
